# Factors Associated With Uptake of Iron Supplement During Pregnancy Among Women of Reproductive Age in Tanzania: an Analysis of Data From the 2015 to 2016 Tanzania Demographic and Health Survey and Malaria Indicators Survey

**DOI:** 10.3389/fpubh.2021.604058

**Published:** 2021-07-14

**Authors:** Fabiola Vincent Moshi, Walter C. Millanzi, Ipyana Mwampagatwa

**Affiliations:** ^1^Department of Nursing Management and Education, School of Nursing and Public Health, University of Dodoma, Dodoma, Tanzania; ^2^Department of Obstetric and Gynecology, School of Medicine and Dentistry, University of Dodoma, Dodoma, Tanzania

**Keywords:** iron, supplement, uptake, pregnancy, factors, Tanzania

## Abstract

**Background:** Pregnant women are vulnerable to iron deficiency due to the fact that more iron is needed primarily to supply the growing fetus and placenta and to increase the maternal red cell mass. Little is known on the factors associated with uptake of iron supplement during pregnancy.

**Methods:** The study used data from the 2015 to 2016 Tanzania Demographic and Health Survey and Malaria Indicators Survey. A total of 6,924 women of active reproductive age from 15 to 49 were included in the analysis. Both univariate and multiple regression analyses were used to determine factors associated with uptake of iron supplement during pregnancy.

**Results:** Majority of the interviewed women 5,648 (81.6%) always took iron supplement during pregnancy, while a total of 1,276 (18.4%) women never took iron supplement during pregnancy. After controlling for confounders, the predictors for uptake of iron supplement during pregnancy were early antenatal booking (adjusted odds ratio, AOR = 1.603 at 95% CI = 1.362–1.887, *p* < 0.001); rural residence (AOR = 0.711 at 95% CI = 0.159–0.526, *p* = 0.007); wealth index [rich (AOR = 1.188 at 95% CI = 0.986–1.432, *p* = 0.07)]—poor was the reference population; level of education [primary education (AOR = 1.187 at 95% CI = 1.013–1.391, *p* = 0.034)]—no formal education was the reference population; parity [para 2 to 4 (AOR = 0.807 at 95% CI = 0.668–0.974, *p* = 0.026), para 5 and above (AOR = 0.75 at 95% CI = 0.592–0.95, *p* = 0.017)], para 1 was the reference population; zones [mainland rural (AOR = 0.593 at 95% CI = 0.389–0.905, *p* = 0.015) and Unguja Island AOR = 0.63 at 95% CI = 0.431–0.92, *p* = 0.017]—mainland urban was the reference population; and current working status [working (AOR = 0.807 at 95% CI = 0.687–0.949, *p* = 0.009)].

**Conclusion:** The study revealed that, despite free access to iron supplement during pregnancy, there are women who fail to access the supplement at least once throughout the pregnancy. The likelihood to fail to access iron supplement during pregnancy was common among pregnant women who initiated antenatal visits late, were from poor families, had no formal education, reside in rural settings, had high parity, were from mainland rural, and were in working status. Interventional studies are recommended in order to come up with effective strategies to increase the uptake of iron supplement during pregnancy.

## Introduction

Iron deficiency among pregnant women remains the most prevalent nutritional problem around the globe because many women enter pregnancy with insufficient iron stores (1). The problem is described in pregnancy as the reduction of the concentration level of hemoglobin below 11 g/dl for the first and third trimester and 10.5 g/dl for the second trimester that occurs in a woman's body during pregnancy and thus restricting the supply in various tissues (1). Iron status during pregnancy is influenced by several factors, including dietary iron intake, iron body stores, adaptive mechanism of the body through the gastrointestinal tract, and the administration of supplements (1). Of the total quantity of stored iron among adult pregnant women, ~3.2 g or 70% of it is contained as hemoglobin, 25% as ferritin which is stored in the spleen, bone marrow, and body fluids, 4–5% as myoglobin, and 1% as intracellular enzymes (1). An iron increase of approximately 1 g (400 mg for the expanded red cell mass, 300–400 mg for fetal hemoglobin, and 100 mg to replace it from bleeding during and after delivery) is required during pregnancy (1). It is estimated that the global prevalence of anemia among women of reproductive age is 29.4%. The prevalence increased with the pregnancy status of a woman, with a global prevalence of 38.4% during pregnancy and 29.0% when not pregnant. There is a global difference in the burden of anemia during pregnancy; only 18% of the global prevalence are from developed countries, and 35–75% of anemia cases during pregnancy are from low- and middle-income countries (2).

Approximately 30% of the global population suffer from iron deficiency anemia, of which most of them live in developing countries (3, 4). The changes in nutrition among pregnant and non-pregnant women occur due to the quality of food than the amount, with the recommendation of eating food containing less fat and carbohydrates but more of protein, calcium, iron, and vitamins. However, it is argued that since the average diet may not adequately meet the pregnancy needs, 30–60 mg of elemental iron should be given daily to supplement the diet beginning on the second and/or third trimester of pregnancy (5, 6). It appears that for elemental iron tablets to be effective, they should be prescribed among all pregnant women despite the diet being considered adequate (7). The non-compliance among pregnant women of prophylactically prescribed iron tablets increases the risk of iron deficiency anemia during pregnancy and the perinatal period.

Iron deficiency anemia among pregnant women has been associated with various maternal and fetal health outcomes (8). Adverse maternal health outcomes may include urinary tract infections, pyelonephritis, pre-eclampsia, hypotension, decreased blood viscosity, thrombotic events, excessive peripertum blood loss, and even deaths. On the other hand, fetal adverse health outcomes from maternal iron deficiency during pregnancy (which is sometimes undetected during birth) include, but are not limited to, low birth weight, pre-term delivery, and premature deaths (4, 9).

Contrary to the advantages of iron supplements among pregnant women and a child in the womb, there have been some arguments from authors that routine iron supplementation during pregnancy has tendencies of impairing zinc absorption and causes gastrointestinal discomforts among pregnant women; furthermore, not all women who are administered with iron supplementation have iron deficiency (5). However, it appears unclear to some people that individual supplementation of iron and improved dietary intake during pregnancy are the best approaches and regimen to help address the problem of maternal and fetal iron deficiency and its associated adverse health outcomes (1, 10).

Compliance to routine iron supplementation among pregnant women has the potential to predict maternal and fetal health during pregnancy and delivery and postnatally. Compliance to routine iron supplementation has been defined by Workineh et al. (11) as taking iron–folic acid tablets for 90 days and above. However, data indicate that the current compliance to iron supplementation among pregnant women is lower than the World Health Organization's recommendation (8, 12). Moreover, findings observed by Fouelifack et al. (13) in their study on the adherence to iron supplementation among pregnant women revealed that, out of 304 recruited women in their study, only 16.4% demonstrated a high compliance with iron supplementation during pregnancy. Nevertheless, a systematic review and meta-analysis findings by Desta et al. (14) showed that only 46.2% of pregnant women adhered to iron supplementation during pregnancy. Based on such findings, non-compliance to routine iron supplementation among pregnant women is still a problem around the globe. A number of approaches have been recommended to avoid and/or solve the problem of non-compliance to iron supplementation among pregnant women, including changing the frequency of iron supplementation from daily to weekly.

The weekly iron supplementation has been proven to be of high efficacy by some authors, including Goshtasebi and Alizadeh (15) and Shankar et al. (16), in randomized controlled trials inclusive of the fortification of iron in foods such as wheat or cow milk. All tested approaches focused on improving the adherence to iron supplementation among pregnant women. In contrast to the efforts done to cater pregnant women's and the growing fetus' health, iron supplementation uptake among women still prevails. This work acknowledges that the immediate predictors to the low uptake of iron supplement among pregnant women considerably depend on their levels of knowledge and attitude about it and its associated health advantages.

On the other hand, other authors (7, 13, 17) link the low uptake of iron supplementation among pregnant women with levels of knowledge, attitude, and skills as demonstrated by health care providers about the benefits of iron supplementation and the recognition of the importance of giving appropriate information and advice among pregnant women. With that note, it appears that what exactly influences the high or low uptake of iron supplementation among women is not clear. Additionally, there seems to be an unanswered question about the topic under study, such as the following: “What influences pregnant women to adhere or not to iron supplementations?,” “Where do pregnant women get advice to adhere and take iron supplementations?,” and or “What is the family's, friends', and health care providers' role on iron supplementation adherence among pregnant women?”

With that in mind, there seems to be no conclusive statement about the exact factors influencing the uptake of iron supplementation among pregnant women. This work intended to address the gap by reviewing and analyzing data from the 2015 to 2016 Tanzania Demographic and Health Survey and Malaria Indicators Survey (2015–2016 TDHS-MIS). The aim was to establish a conclusive statement about the predictors of iron supplement uptake among pregnant women by using existing and published reports and/or studies.

## Materials and Methods

### Study Area and Period

The study was conducted in the United Republic of Tanzania from August 22, 2015 through February 14, 2016. Tanzania is among the countries found in East Africa. It is the largest country that covers 940,000 and 60,000 km^2^ of it is inland water. The country lies south of the equator and shares borders with eight countries as follows: Kenya and Uganda to the north, Rwanda, Burundi, the Democratic Republic of Congo, and Zambia to the west, and Malawi and Mozambique to the south.

### Study Design

It was a national-based cross-sectional study utilizing the 2015–2016 TDHS-MIS dataset.

### Study Population

All women of reproductive age (aged 15–49 years) were included in the study population. The study used individual file records (TZIR7BFL), with a total of 13,266 women who responded to the survey (97% response rate). The study included only women who remembered the timing for antenatal booking of their youngest child. Those who were not able to recall the timing and those who did not respond to the question were removed from the analysis. A total of 6,924 women who had given birth within 5 years preceding the survey were included in the study.

### Sampling Technique

Two stages of sampling were used to obtain a sample for urban and rural areas in Tanzania mainland and Zanzibar. In the first stage, a total of 608 clusters were selected, and in the second stage, a systematic selection of households was involved. A total of 22 households were then systematically selected from each cluster, yielding a representative probability sample of 13,376 households for the 2015–2016 TDHS-MIS.

### Data Collection Tool

The 2015–2016 TDHS-MIS used household questionnaires and individual questionnaires. These questionnaires were based on the Measure DHS standard, AIDS Indicator Survey, and Malaria Indicator Survey questionnaire standards. They were adapted and modified to reflect the Tanzanian population. They were translated into Kiswahili, Tanzania's national language. The data presented in this study are from the individual questionnaires.

### Study Variables

This study's dependent variable is the uptake of iron supplement, whereas the independent variables include socio-demographic characteristics (maternal age, maternal education, place of residence, wealth index, marital status, and parity).

### Data Analysis

The Statistical Package for Social Sciences (IBM SPSS, version 20) was used to analyze the extracted data from TDHS-MIS. The process started by describing all study variables using frequencies and percentages. We then assessed the association between dependent variables and independent variables using the chi-square test, and, finally, we performed binary logistic regression analysis (univariate and multivariate) to determine significant predictors of uptake of iron supplement. All analyses were based on a 5% level of significance.

## Results

Majority of the study respondents (5,113, 73.8%) resided in the rural setting of Tanzania, were of ages 20 to 34 years (4,557, 65.8%), had primary education (4,209, 60.8%), and were married (5,650, 86.1%; Table 1).

**Table 1:** Socio-demographic characteristics.

**Variables**	**Frequency**	**Percent (%)**
**Place of residence**		
Urban	1,811	26.2
Rural	5,113	73.8
**Age group**		
Less than 20 years	541	7.8
20–34 years	4,557	65.8
More than 34 years	1,826	26.4
**Educational level**		
No education	1,329	19.2
Primary education	4,209	60.8
Secondary	1,326	19.2
Higher	60	0.9
**Parity**		
Para 1	1,595	23
Para 2–4	3,154	45.6
Para 5+	2,175	31.4
**Wealth index**		
Poor	2,734	39.5
Middle	1,363	19.7
Rich	2,827	40.8
**Respondent currently working**		
Not working	1,498	21.6
Working	5,426	78.4
**Mainland/Zanzibar**		
Mainland urban	1,618	23.4
Mainland rural	4,357	62.9
Unguja (Zanzibar Island)	594	8.6
Pemba (Pemba Island)	355	5.1

### The Relationship Between Women's Characteristics and Uptake of Iron Supplement

The women's characteristics which showed a significant relationship with uptake of iron supplement were timing for antenatal booking (*p* < 0.001), age group of a woman (*p* = 0.002), educational status (*p* < 0.001), parity (*p* < 001), wealth index (*p* = 0.001), and working status (*p* = 0.005; Table 2).

**Table 2:** The relationship between women's characteristics and uptake of iron supplement.

**Variables**	**Given or bought iron tablets/syrup**		***X*^**2**^**	***p*-value**
	**No, *n* (%)**	**Yes, *n* (%)**		
**Antenatal booking**				
Early booking	206 (13)	1,377 (86.8)		
Late booking	1,070 (20)	4,266 (79.9)	44.084	<0.001
**Place of residence**				
Urban	313 (17.3)	1,495 (82.6)		
Rural	963 (18.8)	4,148 (81.1)	5.044	0.08
**Age group**				
Less than 20 years	100 (18.5)	441 (81.5)		
20–34 years	783 (17.2)	3,771 (82.8)		
More than 34 years	393 (21.5)	1,431 (78.4)	17.168	0.002
**Educational level**				
No education	295 (22.2)	1,032 (77.7)		
Primary education	758 (18)	3,449 (81.9)		
Secondary	215 (16.2)	1,111 (83.8)		
Higher	8 (13.3)	51 (85)	42.037	<0.001
**Parity**				
Para one	241 (15.1)	1,353 (84.8)		
Para 2–4	567 (18)	2,586 (82)		
Para 5+	468 (21.5)	1,704 (78.3)	28.096	<0.001
**Wealth index**				
Poor	568 (20.8)	2,164 (79.2)		
Middle	238 (17.5)	1,125 (82.5)		
Rich	470 (16.6)	2,354 (83.3)	18.401	0.001
**Respondent currently working**				
Not working	234 (15.6)	1,264 (84.4)		
Working	1,043 (19.2)	4,379 (80.7)	10.525	0.005
**Mainland/Zanzibar**				
Mainland urban	272 (16.5)	1,350 (83.4)		
Mainland rural	835 (19.2)	3,520 (80.8)		
Unguja (Zanzibar Island)	112 (18.90)	482 (81.1)		
Pemba (Pemba Island)	64 (18)	291 (82)	10.068	0.122

### Predictors of Uptake of Iron Supplement

After controlling for confounders, the predictors for uptake of iron supplement during pregnancy were early antenatal booking (adjusted odds ratio, AOR = 1.603 at 95% CI = 1.362–1.887, *p* < 0.001), rural residence (AOR = 0.711 at 95% CI = 0.159–0.526, *p* = 0.007), wealth index [rich AOR = 1.188 at 95% CI = 0.986–1.432, *p* = 0.07)]—poor being the reference population, level of education [primary education (AOR = 1.187 at 95% CI = 1.013–1.391, *p* = 0.034)]—no formal education was the reference population, parity [para 2–4 (AOR = 0.807 at 95% CI = 0.668–0.974, *p* = 0.026), para 5 and above (AOR = 0.75 at 95% CI = 0.592–0.95, *p* = 0.017)]—para one was the reference population, and zones [mainland rural (AOR = 0.593 at 95% CI = 0.389–0.905, *p* = 0.015), Unguja Island (AOR = 0.63 at 95% CI = 0.431–0.92, *p* = 0.017]—mainland urban was the reference population, and current working status [working (AOR = 0.807 at 95% CI = 0.687–0.949, *p* = 0.009)] (Table 3).

**Table 3:** Predictors of uptake of iron supplement.

**Variable**	**OR**	**95% CI**		***p*-value**	**AOR**	**95% CI**		***p*-value**
		**Lower**	**Upper**			**Lower**	**Upper**	
**ANC booking**								
Late booking	1				1			
Early booking	1.679	1.43	1.973	<0.001	1.603	1.362	1.887	<0.001
**Place of residence**								
Urban					1			
Rural	0.9	0.782	1.036	0.144	0.711	0.159	0.526	0.007
**Age groups**								
Less than 20 years	1				1			
20 to 34 years	1.093	0.868	1.376	0.449	1.216	0.936	1.581	0.143
More than 34 years	0.827	0.648	1.055	0.127	1.05	0.77	1.432	0.758
**Wealth index**								
Poor	1				1			
Middle	1.24	1.048	1.466	0.012	1.173	0.988	1.393	0.069
Rich	1.315	1.149	1.506	<0.001	1.188	0.986	1.432	0.07
**Educational level**								
No education	1				1			
Primary education	1.299	1.116	1.511	0.001	1.187	1.013	1.391	0.034
Secondary	1.474	1.213	1.792	<0.001	1.207	0.957	1.524	0.112
Higher	1.854	0.871	3.948	0.109	1.374	0.632	2.989	0.423
**Parity**								
Para 1	1				1			
Para 2–4	0.812	0.689	0.957	0.013	0.807	0.668	0.974	0.026
Para 5+	0.649	0.547	0.77	<0.001	0.75	0.592	0.95	0.017
**Current working status**								
Not working	1				1			
Working	0.774	0.663	0.904	0.001	0.807	0.687	0.949	0.009
**Mainland/Zanzibar**								
Mainland urban	1				1			
Mainland rural	0.826	0.71	0.962	0.014	0.593	0.389	0.905	0.015
Unguja (Zanzibar Island)	0.843	0.66	1.076	0.17	0.63	0.431	0.92	0.017
Pemba (Pemba Island)	0.891	0.659	1.203	0.45	0.694	0.441	1.09	0.112

## Discussion

Majority of the study respondents were between 20 years and 34 years, with a few >34 years. Most of them were residing in rural settings of Tanzania, whereas very few were from Zanzibar (Unguja and Pemba). The findings showed that majority of the interviewed women (81.6%, *n* = 5,648) always took iron supplement during pregnancy, while 18.4% (*n* = 1,276) of the women never took iron supplement during pregnancy. There was a strong association between the educational level of the study respondents and adherence to iron supplementation, in which as one advanced in education, she was more times likely to adhere to iron supplementation than those who had never passed through formal education. This has been a case in this study; educated women seemed to be exposed to and able to search information about the advantages of using iron supplements during pregnancy than their uneducated counterparts. Such findings concur with other studies done in India, for example, by Manasa et al. (18) who uncovered through their findings that the level of education of an individual increases awareness and understanding on the importance of micronutrients for good fetal development. Thus, educational level has become a potential positive predictor on iron supplement uptake among pregnant women.

Nevertheless, early antenatal booking is indicated to be strongly associated with iron supplement uptake among the study respondents in the study. The findings revealed that as pregnant women book antenatal clinics as early as possible, the higher they adhere to iron uptake. This could be the case because the more pregnant women book for an antenatal clinic, the more they access antenatal information and its associated services. These findings tally with those by Urassa et al. (19) who reported that early antenatal booking among pregnant women is a significant predictor to their levels of adherence and uptake of iron supplementation. Good adherence to iron supplementation among pregnant women has always been associated with the good growth and development of the fetus in the womb.

Moreover, the findings of this study exposed that the residence of respondents was strongly associated with iron supplement uptake among pregnant women, in which it was observed that living in a rural area was a negative predictor to iron uptake. This would be the case because people living in urban areas would benefit with the consistent accessibility to information about the benefits of micronutrients, their availability, and motive to use them during pregnancy. The existence of disparities in access to maternal health services between rural and urban dwellers could have contributed to a negative prediction on the uptake of iron supplements among pregnant women residing in rural areas in Tanzania. In urban settings, there are several maternal and child health centers (both private and government) where these women can access maternal and child health services. The situation is different in the rural settings where, in some rural areas, their health facilities are situated far from their homes, with seasonal roads and poor means of transport. The availability of supplies could also explain the low uptake of iron supplements among pregnant women residing in rural areas if compared to those in urban areas. The rate of out-of-stock of iron supplement is far higher in rural settings if compared to urban settings. The findings match those by Zavaleta et al. (20) who noted that pregnant women living in rural areas were less likely to adhere to iron supplementation as most of the time they are not easily updated on its benefits to the growing fetus and sometimes suffer from the non-availability and inaccessibility of iron supplements.

The findings of this study indicated that the parity level of pregnant women was strongly associated with the uptake of iron supplements. It was found that, para 1 pregnant women were more likely to adhere to iron supplementation than women with para 2 and above. This could be discussed in the current study as attributed to the reduced perceived benefits (neglect behavior) that would be demonstrated later by the pregnant women. A para 1 woman could take it seriously because it was her first time to be exposed to such management, and therefore it could be easy for them to adhere to the antenatal services provided to them. Another reason could be due to low risk perception toward pregnancy and childbirth. A previous qualitative study done in rural Tanzania has revealed that multiparity women with uneventful pregnancy and childbirth tend to perceive pregnancy and childbirth as a normal process which is not associated with problems (21). This perception contributes greatly to their maternal services utilization. Such findings were also observed by Workineh et al. (11) who reported that the more a woman delivers children, the more she does not comply to iron supplementation.

The working status of the respondents demonstrated a strong association with the uptake of iron supplements among them. It was found that pregnant women who were not working adhered to iron supplementation compared to those having been employed or self-employed. These findings imply that pregnant women with no job to do could have time, adhere to the appointments of attending clinics as scheduled, and therefore take iron supplements than pregnant women who were working. Such findings are supported by Kiwanuka et al. (22) whose findings of the study revealed that having been employed or self-employed minimizes the time to attend antenatal clinics as scheduled and thus affects the uptake of iron among pregnant women.

Additionally, the wealth index of the respondents was strongly associated with their iron supplement uptake. The findings showed that pregnant women with middle and rich wealth index were less likely to adhere to iron supplementation uptake than their poor counterparts. This implies that people with higher wealth index might perceive that they can buy and use other remedies in place of iron supplements than poor people could do. On the other hand, free antenatal health information and its associated services provided among people *via* health facilities in the country could even motivate poor people to access and use them as scheduled than if they could be sold. In contrast, Ba et al. (17) reported that higher proportions of pregnant women with low economic status were not complying with iron supplementation. However, this could be discussed as due to the geographical and political differences between these studies.

However, the age groups of the respondents was not observed to be among the predictors of iron supplement uptake among the study respondents. The study found higher odds as the age of the respondents increased, but there was no significant association. These findings contradict those by Boti et al. (23) who found age to be a predictive factor to adherence to iron supplementation. The explanation to this could be due to the fact that the latter study was conducted in a developed country where access to information among all age groups is readily available, and thus their literacy level is much higher.

### Strengths of the Study

Responding to sustainable development goal number 3, target number 3.2, of ending all preventable morbidity and mortality rates among women of reproductive age and newborns, this paper has addressed a very important topic to reveal unclear information about the uptake of iron supplements during pregnancy. Nevertheless, the paper has drawn and analyzed the data of a nationally representative survey from a demographic and health study (DHS) which has a high response rate and wide national coverage and therefore gives a reliable and high-probability estimate of the impact of iron supplementation during pregnancy.

### Limitation of the Study

Being a review of DHS data, there might be some degree of recall bias during data collection, though not to a high degree. Additionally, the methods used in this paper might not be sufficiently documented to allow replication studies.

## Conclusion

The study revealed that, despite of free access to iron supplements during pregnancy, there are women who fail to access the supplement at least once throughout their pregnancy. The likelihood to fail to access the iron supplements during pregnancy was higher among pregnant women who initiated antenatal visits late, were from poor families, had no formal education, reside in rural settings, had high parity, were from mainland rural, and were in a working status. Interventional studies are recommended in order to come up with effective strategies to increase the uptake of iron supplement during pregnancy.

## Data Availability Statement

The original contributions presented in the study are included in the article/supplementary material, further inquiries can be directed to the corresponding author/s.

## Ethics Statement

The procedures for DHS-MIS data collection were approved by the following organizations: Tanzania's National Institute for Medical Research (NIMR), the Zanzibar Medical Ethics and Research Committee (ZAMREC), the Institutional Review Board of ICF International, and the Centers for Disease Control and Prevention in Atlanta, USA. Written informed consent to participate in this study was provided by the participants' legal guardian/next of kin.

## Author Contributions

FM conceptualized the study, drafted the method section, did the analysis of the secondary data, and critically reviewed the final copy of the manuscript. WM wrote the introduction, discussed the findings, and critically reviewed the manuscript. IM directed the conceptualization and critically reviewed the manuscript. All the authors approved the submission of this manuscript.

## Conflict of Interest

The authors declare that the research was conducted in the absence of any commercial or financial relationships that could be construed as a potential conflict of interest.
